# Dynamic analysis of cylindrical foundations under torsional loading via generic discrete-element models simulating soil stratum

**DOI:** 10.1038/s41598-023-46046-7

**Published:** 2023-11-06

**Authors:** Shi-Shuenn Chen, Chi-Jou Kao, Jun-Yang Shi

**Affiliations:** 1 Department of Civil and Construction Engineering, National Taiwan University of Science and Technology, Taipei, 106 Taiwan; 2 Department of Civil and Environmental Engineering, National University of Kaohsiung, Kaohsiung, 811 Taiwan

**Keywords:** Engineering, Civil engineering

## Abstract

Torsional vibration, considering soil-structure interaction, is essential to the dynamic response of most irregular structures. A systematic method is developed to seek the optimal simplified model among multiple model candidates for uniform soil on rigid base regarding dynamic soil-foundation interactions. A generic model is identified by the proposed method to simulate the cylindrical foundation resting on or embedded in the soil stratum under torsional vibrations. Various soil-foundation parameters, mainly including embedment depth, layer depth, and mass ratios, are considered in the simplified analysis. The frequency-magnification curves and resonant responses of the foundation using the generic model agree well with theoretical solutions. The resultant resonant magnification factors against mass ratios clearly illustrate the impacts of the whipping effect resulting from the soil-foundation interactions. The generic model performs better and adopts fewer parameters than the existing model to simulate the soil-foundation interactions. In addition, dimensionless parametric charts are presented to estimate foundation responses for engineering applications quickly. The proposed charts also significantly overcome the limitations of the Wolf and Paronesso model. The generic model shows efficiency and accuracy in simulating the soil stratum. This research could contribute to the foundation vibration analysis for torsional responses.

## Introduction

Research on soil dynamics and structures has developed many fundamental methods for formulating interaction problems. The causes of the torsional response of buildings are mostly related to the eccentric distribution of stiffness, damping, and the mass of a structure, or torsional excitations^1–9^. The torsional response considering SSI is a key issue to be concerned about, as the rotation can significantly contribute to the response of most buildings, bridges, and steel structures during earthquakes^10–12^. Complex engineering problems can be solved by developing simplified models. Deifalla and Mukhtar^13^ constructed a simplified model for reinforced concrete elements under combined shear and axial tension. Fu et al.^14^ utilized the detachment phenomenon to create a mechanical model of pipe-soil interaction for pipe deformation under tunnel excavation. Cui et al. investigated the analytical solutions of the pile-soil interaction behavior in a uniform soil on bedrock or layered soils under vertical vibrations^15,16^. Wang et al.^17^ developed a 3D finite-element model to investigate the seismic performance of large-scale pipeline structures under multiple external forces. To analyze dynamic SSI problems, the evaluation of dynamic impedance functions (i.e. stiffness and damping) plays a key role due to the frequency-dependent characteristics of the impedance function^18^. Numerous lumped-parameter models simulate SSI for foundations in a uniform half-space under torsional vibrations^19–24^. Besides the simulation for a uniform half-space, some research considering foundations in layered soils was also investigated. Wolf and Somaini^20^ executed a five-parameter discrete model to analyze the torsional vibrations of a square foundation on layered soils with linearly varying shear-wave velocities. Wolf and Paronesso^25^ presented an eight-parameter model with three degrees of freedom (DOFs) for a cylindrical foundation vibrating in horizontal, vertical, rocking, or torsional motions, overlaying and embedded in a uniform soil layer on a rigid base. Pana et al.^26^ adopted an imperfect interface bonding model to study the vertical and torsional vibrations of a rigid circular disc on a transversely isotropic half-space. Shi^27^ presented a systematic modeling approach for layered soils considering rotational and horizontal foundation excitations. Dynamic responses of the foundation matched theoretical and computer-based solutions. Shi et al.^28^ developed a model with three or five elements to simulate square foundations embedded in a nonuniform layer undergoing vertical load. In addition, Shi et al.^29^ also presented an adaptive method containing 33 model candidates to investigate shallow foundations subjected to vertical excitations.

Most past studies concerning vibrational foundation response were limited to simulate dynamic SSI in a homogeneous half-space. Wolf and Somaini^20^ didn’t demonstrate its application for the square foundation in soils overlying rigid bases. The Wolf and Paronesso model^25^ (1992) was a pioneer research but didn’t show the simplicity of modeling elements or provide related application charts. Thus, this article emphasizes using a systematic method of lump-parameter models to adaptively find a simple generic model for a cylindrical foundation in a uniform soil layer on rigid base and further offers numerical values of model parameters for easier and faster practical applications considering a wider range of soil-foundation parameters. Additionally, this article catches resonant responses of the foundations for the discrete frequency–response curves by applying the five-point-interpolation method by Lysmer et al.^30^. The resonant results are then compared with those from rigorous solutions and a corresponding existing model by Wolf and Paronesso^25^. It is worth mentioning that the main differences between this research and previous related studies are demonstrated in Table 1. This research aims to investigate the torsional SSI behavior of uniform soil using a systematic method with only seven model candidates. Graphical charts are also presented to determine the model parameters. In addition, the determination of model parameters is independent of mass ratios. In this paper, the theoretical background of the proposed method is demonstrated first. The model selection for the target soil-foundation system is then illustrated. Subsequently, the model validations on frequency–response curves and resonant responses are investigated. A multiple target approach for practical applications is provided in the next section. A conclusion is made in the last section. This paper may bring extensive insight and efficiency for uniform soil on rigid base in the torsional vibration analysis of foundations.

**Table 1:** Comparison of previous related research by the authors.

Research work	Vibration mode	Model candidates	Model limitation for vibrating mass
Present study	Torsional mode	7	None (Independent of mass ratios)
Ref.^27^	Horizontal and rocking modes	276	Depend on mass ratios
Ref.^28^	Vertical mode	2	None (Independent of mass ratios)
Ref.^29^	Vertical mode	33	Depend on mass ratios

## Developed systematic method

**Figure 1:**
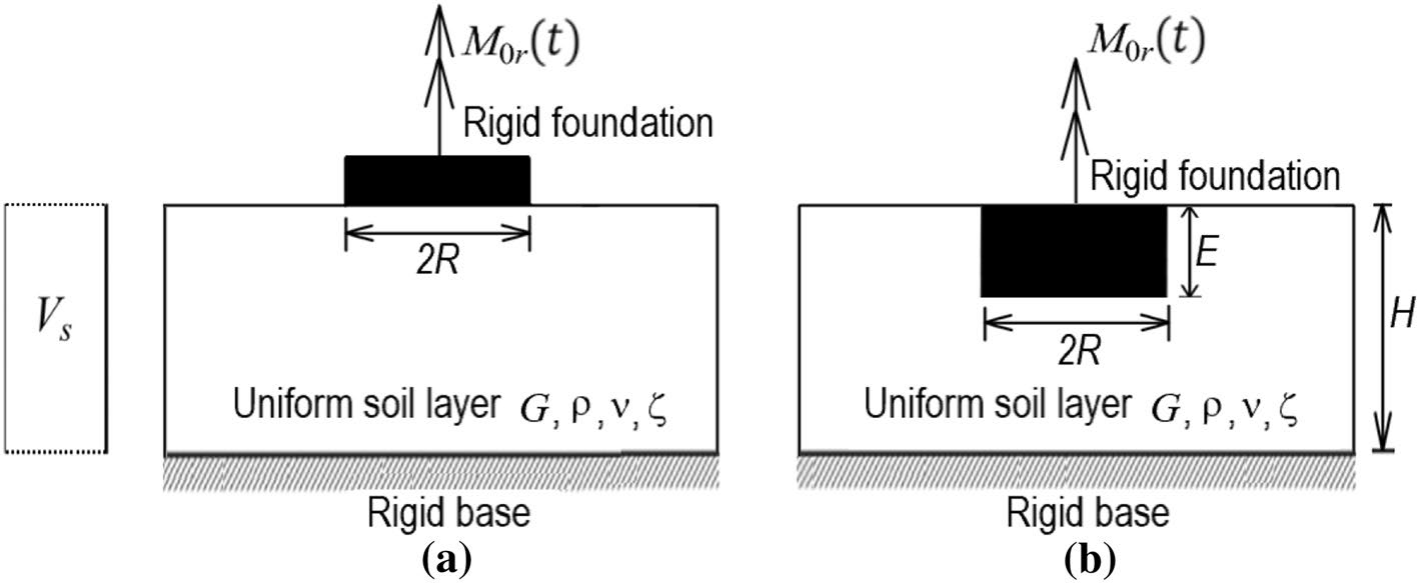
Soil-foundation system subjected to torsional vibration. (**a**) cylindrical resting on uniform soil on rigid base, (**b**) cylindrical embedded in uniform soil on rigid base.

This section shows a systematic method to generate lumped-parameter models and to find the optimal model to simulate a cylindrical foundation on a uniform stratum undergoing harmonic torsional force. The soil stratum is viscoelastic and entirely connected to the cylindrical foundation overlaying or embedded in a homogenous soil layer on a rigid base, as shown in Fig. 1a,b. The cylindrical foundation that has a radius *R* is overlaying and embedded in a soil layer on rigid base, subjected to a harmonic torque $M_{0r}$. The target soil condition is considered to be a uniform stratum on a rigid base with a depth of *H* where the shear-wave velocity, $V_{s}$, is uniformly distributed; the Poisson ratio, *ν*, is 0.33; the damping ratio, *ζ*, is 0.05; *G* is the shear modulus; *E* is the embedded depth of a foundation. ρ is the soil mass density and $V_{s}$ is the shear-wave velocity of the soil. This research considers the SSI behavior using a foundation impedance function describing the force–displacement relation at the interface between the soil and the foundation. Note that the foundation represents one part of the upper structures.

For parametric analysis, $a_{0} = \omega R/V_{s}$ is the dimensionless frequency of harmonic torsional force with forcing frequency $\omega$; *T* is defined as embedment ratio (i.e., *T* = *E*/*R*); *D* is defined as layer depth ratio (i.e., *D* = *H*/*R*); $b$ is the dimensionless mass ratio for torsional motion, (i.e., mass ratio in short in the following of the paper) and is non-dimensionalized as $b = I_{r} /\rho R^{5}$, where $I_{r}$ is the polar mass moment of inertia of the foundation around the axis of rotation. It is noted that the polar mass moment of inertia is applied as a mechanism for quantifying the soil-foundation interaction.

### Model candidates

**Figure 2:**
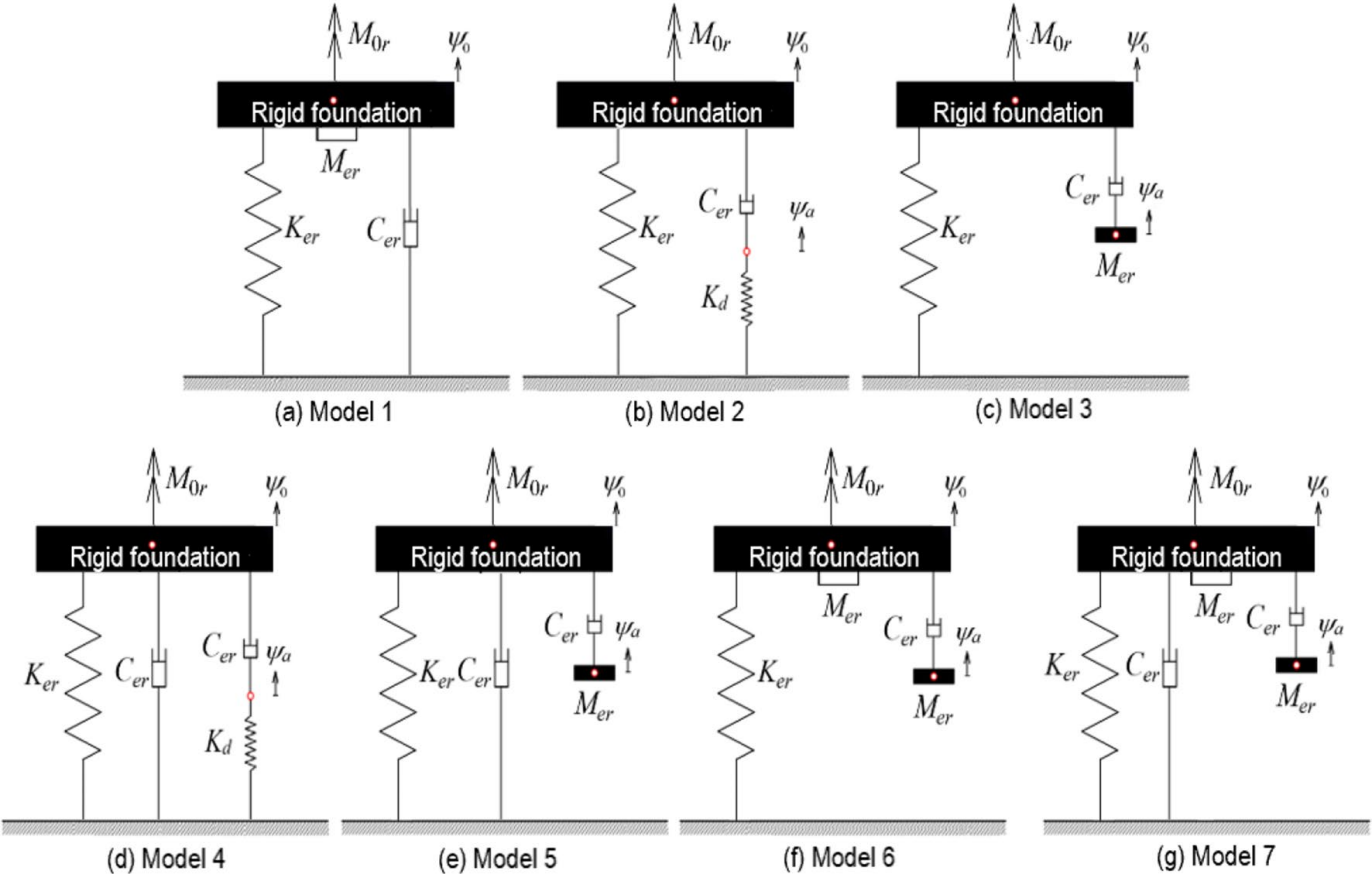
Proposed simplified models with seven candidates.

The systematic method comprises seven simplified models, which are developed to simulate the uniform soil on a rigid base subjected to torsional vibration, as shown in Fig. 2. The simplified models are arranged sequentially from Model 1 to Model 7, as shown in Fig. 2a–g. Each of the seven simplified models connects to the foundation and the rigid base. The maximum internal degree of freedom is two for all simplified models where *ψ*_0_ is the rotation of the foundation about the vertical axis of symmetry and *ψ*_*a*_ is the additional rotational DOF about vertical axis. The simplified models are developed to simulate the relationship of the soil-foundation interaction behavior. The modeling elements of the proposed models include lumped masses moment of inertia, viscous dampers, and linear springs. The model parameters contain static torsional stiffness,* K*_*er*_, dynamic torsional stiffness,* K*_*d*_, the polar mass moment of inertia,* M*_*er*_, and torsional damping, *C*_*er*_. Each simplified model considers only three parameters:* K*_*er*_, *C*_*er*_, and either* K*_*d*_ or *M*_*er*_. The layout of each proposed model is summarized as follows: Model 1 consists of three elements connected in parallel. Model 2 has three elements and one internal DOF contributed by a serial connection of a spring and a damper. Similarly, Model 3 also has three elements and one internal DOF given by a damper connected with a polar mass moment of inertia. Model 4 and Model 5 include four elements and one internal DOF. They are featuring in having two torsional dampers. Model 6 and Model 7 consist of four and five elements, respectively, while two polar masses moment of inertia are included in their layout. The parameters of the proposed models could be non-dimensionalized as follows:

1 $$k_{er} = \frac{{K_{er} }}{{GR^{3} }},\; k_{d} = \frac{{K_{d} }}{{GR^{3} }},\;m_{er} = \frac{{M_{er} }}{{\rho R^{5} }},\; c_{er} = \frac{{C_{er} }}{{\rho V_{s} R^{4} }}$$

The systematic method calculates the parameters of simplified models using equivalent principles for static and dynamic responses. The adoption of equivalent theories is based on equaling the dynamic impedance of unbounded soils to the torsional impedance function of the simplified models; thus, it is assumed that the cylindrical foundation is massless. The torsional impedance of a target soil-foundation system is used in this study to calculate the model parameters for equivalent models. Based on the vibration theory, when each simplified model is subjected to a harmonic torque, the torsional impedance function of each model is defined as follows:

2 $$\overline{K}_{r} = \overline{K}_{sr} \left( {\overline{k}_{r} + ia_{0} \overline{c}_{r} } \right)$$

where $\overline{K}_{sr}$ is the static stiffness; $\overline{k}_{r}$ and $\overline{c}_{r}$ are the dynamic stiffness and damping coefficients, respectively.

The systematic method proposed uses three equivalent criteria to calculate the model parameters. Assume the torsional impedance function of a soil-foundation system is $K_{r} = K_{sr} (k_{r} + ia_{0} c_{r} )$. By equalizing the impedance function of a soil-foundation system to that of a simplified model, the equivalent equations regarding static stiffness, dynamic stiffness coefficients, and dynamic damping coefficients are established as follows:

3 $$\overline{K}_{sr} = K_{sr}$$

4 $$\overline{k}_{r} = k_{r}$$

5 $$\overline{c}_{r} = c_{r}$$

where $K_{sr}$, $k_{r}$, $c_{r}$ are the static stiffness, the dynamic stiffness coefficients, and the dynamic damping coefficients, respectively, for the target soil-foundation system subjected to torsional vibration. The first equivalent equation adopts the static equilibrium of a soil-foundation system. Therefore, the static stiffness $K_{er}$ of each model is derived by Eq. (3), i.e.,

6 $$K_{er} = K_{sr}$$

Besides, Table 2 shows the impedance function of each simplified model, which is derived from dynamic equilibrium equations based on structural dynamics. A system of nonlinear equations (SNE) is established by substituting $\overline{k}_{r}$ and $\overline{c}_{r}$ of a model into Eqs. (4) and (5). The other model parameters are further obtained by solving the SNE in this study.

**Table 2:** Dynamic impedance coefficients of the proposed simplified models.

Model no	Stiffness coefficient, $\overline{k}_{r}$	Damping coefficient, $\overline{c}_{r }$
1	$1 - \frac{{a_{0}^{2} m_{er} }}{{k_{er} }}$	$\frac{{c_{er} }}{{k_{er} }}$
2	$1 + \frac{{c_{er}^{2} k_{d} a_{0}^{2} }}{{k_{er} (k_{d}^{2} + c_{er}^{2} a_{0}^{2} )}}$	$\frac{{c_{er} k_{er} }}{{k_{er}^{2} + c_{er}^{2} a_{0}^{2} }}$
3	$1 - \frac{{c_{er}^{2} m_{er} a_{0}^{2} }}{{k_{er} (c_{er}^{2} + m_{er}^{2} a_{0}^{2} )}}$	$\frac{{c_{er} m_{er}^{2} a_{0}^{2} }}{{k_{er} (c_{er}^{2} + m_{er}^{2} a_{0}^{2} )}}$
4	$1 + \frac{{c_{er}^{2} k_{d} a_{0}^{2} }}{{k_{er} (k_{d}^{2} + c_{er}^{2} a_{0}^{2} )}}$	$\frac{{c_{er} }}{{k_{er} }} + \frac{{c_{er} k_{er} }}{{k_{er}^{2} + c_{er}^{2} a_{0}^{2} }}$
5	$1 - \frac{{c_{er}^{2} m_{er} a_{0}^{2} }}{{k_{er} (c_{er}^{2} + m_{er}^{2} a_{0}^{2} )}}$	$\frac{{c_{er} }}{{k_{er} }} + \frac{{c_{er} m_{er}^{2} a_{0}^{2} }}{{k_{er} (c_{er}^{2} + m_{er}^{2} a_{0}^{2} )}}$
6	$1 - \frac{{a_{0}^{2} m_{er} }}{{k_{er} }} - \frac{{c_{er}^{2} m_{er} a_{0}^{2} }}{{k_{er} (c_{er}^{2} + m_{er}^{2} a_{0}^{2} )}}$	$\frac{{c_{er} m_{er}^{2} a_{0}^{2} }}{{k_{er} (c_{er}^{2} + m_{er}^{2} a_{0}^{2} )}}$
7	$1 - \frac{{a_{0}^{2} m_{er} }}{{k_{er} }} - \frac{{c_{er}^{2} m_{er} a_{0}^{2} }}{{k_{er} (c_{er}^{2} + m_{er}^{2} a_{0}^{2} )}}$	$\frac{{c_{er} }}{{k_{er} }} + \frac{{c_{er} m_{er}^{2} a_{0}^{2} }}{{k_{er} (c_{er}^{2} + m_{er}^{2} a_{0}^{2} )}}$

### Frequency-response curves

This subsection aims to illustrate the frequency–response curve of a dynamic system. As the target soil-foundation system shown in Fig. 1 is subjected to a dynamic load, the equation of motion is expressed as follows:

7 $$I_{r} \ddot{\psi }_{0} (t) + K_{r} \psi_{0} (t) = M_{0r} (t)$$

where $M_{0r}$ is the harmonic torque, $I_{r}$ is the polar mass moment of inertia of the foundation around the axis of rotation, $K_{r}$ is the torsional impedance function of the soil-foundation system. Recall that the torsional impedance function is given by $K_{r} = K_{sr} (k_{r} + ia_{0} c_{r} )$. Equation (7) can be used to derive the steady-state responses of the foundation by assuming $M_{0r} = m_{0r} \exp ({\text{i}}\omega t)$ and $\psi_{0} (t)$ = $\phi_{0} \times \exp ({\text{i}}\omega t)$, as shown below.

8 $$\psi_{0} (t) = \frac{{m_{0r} }}{{K_{sr} }}M_{r} \times \exp \left[ {{\text{i}}\left( {\omega t + \theta_{r} } \right)} \right]$$

9 $$M_{r} = 1/\sqrt {\left( {k_{r} - a_{0}^{2} b/k_{sr} } \right)^{2} + \left( {a_{0} c_{r} } \right)^{2} }$$

where $m_{0r}$ is the amplitude of the harmonic torque; $M_{r}$ and $\theta_{r}$ are the dynamic magnification factor and the phase angle.

It is noted that the damped soil-foundation system considers the effect of both material damping and radiation damping on the foundation response. In detail, the effect of the material damping and radiation damping are respectively considered in the real and imaginary part of the impedance function for the target soil-foundation system, as shown in Fig. 1. The theoretical impedances by Tassoulas and Kausel^31^. are adopted for the surface foundation at the depth (*D* = 2) as the corresponding frequency *a*_*0*_ = 0.1 to 6.2 with an interval of 0.1. On the other hand, the theoretical impedances by Tassoulas^32^ are adopted for the foundation embedded at the depth (*T* = 1), layer depth ratios (*D* = 2, and 3) as the corresponding frequency *a*_*0*_ = 0.1 to 3 with an interval of 0.1. Similarly, when using the proposed models (i.e., Model 1 to Model 7) to simulate the soil-foundation system, the dynamic magnification factor of the foundation could be written as follows:

10 $$\overline{M}_{r} = 1/\sqrt {\left( {\overline{k}_{r} - a_{0}^{2} b/\overline{k}_{sr} } \right)^{2} + \left( {a_{0} \overline{c}_{r} } \right)^{2} }$$

where the impedance coefficients $\overline{k}_{r}$ and $\overline{c}_{r}$ are given in Table 2. Therefore, the magnification factor of all simplified models can be calculated by Eq. (10) at each dimensionless frequency for a given mass ratio.

### Optimal model by a single target approach (STA)

A single target approach (STA) is illustrated to find an optimal model for a target soil-foundation system regarding an individual mass ratio. At first, the parameters of the 7 model candidates are calculated at each dimensionless frequency. As there are *NF* dimensionless frequencies, the systematic method establishes 7 × *NF* simplified models. An error function $\varepsilon_{r}$ is defined as follows to search for the optimal equivalent model that reproduces the most accurate frequency–response curve.

11 $$\varepsilon_{r} = \sqrt {\mathop \sum \limits_{i = 1}^{NF} \left( {M_{r} - \overline{M}_{r} } \right)_{i}^{2} \times P_{i} }$$

where $M_{r}$ is the dynamic magnification factor of the target system given by Eq. (9); $\overline{M}_{r}$ is the dynamic magnification factor calculated from the model parameters; $P_{i}$ is the weighting at the $ith$ frequency point and $P_{i} = (M_{r} )_{i}$. Once the impedance functions of a soil-foundation system are given, the three model parameters for each model candidates are directly calculated from Eqs. (4)–(6), and the optimal model with the least value of error function is to be found by implementing Eq. (11). In brief, the procedure of STA considering single mass ratio is summarized as follows:

1. Find the torsional impedance function for a target soil-foundation system.
2. Use Eq. (6) to compute the static stiffness of simplified models.
3. Use Eqs. (4) and (5) to solve the other model parameters. Subsequently, 7 × *NF* simplified models will be constructed for *NF* frequency points.
4. Run an error analysis using Eq. (11) for each model and determine the optimal model with the minimum error.

## A generic model for simulating the target soil-foundation system

This section pays attention to selecting the optimal equivalent model based on the procedure of STA among seven simplified models, which simulate the soil-foundation system in Fig. 1. According to the analyzed results for single mass ratio, Tables 3 and 4 summarizes the error index of all simplified models regarding various simulating conditions. A total of 63 analyzed cases are conducted with different embedment depth ratios (*T* = 0, and 1), layer depth ratios (*D* = 2, and 3), and mass ratios (*b* = 1, 5, and 10). It is observed in Table 3 for surface foundations that optimal simplified models are selected to investigate the target stratum. Model 3 could outperform almost the rest of the six model candidates for the analyzed cases as mass ratio *b* is smaller than 10. It is noted that in the case of mass ratio *b* equaling 10, a minimal discrepancy of error index is found between two model candidates (Model 3 and Model 1). On the other hand, it is observed clearly in Table 4 for the embedded cylindrical foundations that one unique model candidate (Model 3) is consistently calculated to investigate the target stratum, it outperforms the other six model candidates for the analyzed cases of mass ratio, *b*, is smaller than and equal to 10. Based on the numerical analysis of the error index, a conclusion is made that Model candidate (Model 3) is the generic model to simulate the target sol-foundation system as the mass ratio of the foundation is smaller than 10.

**Table 3:** Error index of simplified models for surface cylindrical foundations.

Simulation condition			Model candidates	Dimensionless parameters				Error index
*T* = *E*/*R*	*D* = *H*/*R*	*b*		*k*_*er*_	*k*_*d*_	*c*_*er*_	*m*_*er*_	*ε*_*r*_
0	2	1	Model 1	5.79	–	1.13	0.41	1.735
0	2	1	Model 2	5.79	− 4.93	1.58	–	1.902
0	2	1	Model 3	5.79	–	1.50	1.29	**0.355**
0	2	1	Model 4	5.79	− 0.84	1.36	–	8.881
0	2	1	Model 5	5.79	–	0.30	0.30	2.830
0	2	1	Model 6	5.79	–	0.93	0.89	68.386
0	2	1	Model 7	5.79	–	0.99	0.22	1.724
0	2	5	Model 1	5.79	–	0.58	1.02	5.568
0	2	5	Model 2	5.79	− 1.35	2.37	–	2.531
0	2	5	Model 3	5.79	–	2.37	1.35	**1.699**
0	2	5	Model 4	5.79	− 0.43	1.45	–	385.544
0	2	5	Model 5	5.79	–	0.71	0.31	178.028
0	2	5	Model 6	5.79	–	0.93	0.89	36.521
0	2	5	Model 7	5.79	–	0.30	0.93	4.542
0	2	10	Model 1	5.79	–	0.10	1.09	**0.053**
0	2	10	Model 2	5.79	− 0.54	6.25	–	2.839
0	2	10	Model 3	5.79	–	6.25	1.11	0.059
0	2	10	Model 4	5.79	0.73	0.00	–	1583.259
0	2	10	Model 5	5.79	–	0.70	0.57	1523.450
0	2	10	Model 6	5.79	–	0.10	1.08	0.062
0	2	10	Model 7	5.79	–	0.05	1.09	0.055

*T = *E*/*R* is an embedment ratio, *D* = *H*/*R* is a layer depth ratio, *b* is a mass ratio. N/A indicates no real number solution is found.

Significant values are in bold.

**Table 4:** Error index of simplified models for embedded cylindrical foundations.

Simulation condition			Model candidates	Dimensionless parameters				Error index
*T* = *E*/*R*	*D* = *H*/*R*	*b*		*k*_*er*_	*k*_*d*_	*c*_*er*_	*m*_*er*_	*ε*_*r*_
1	2	1	Model 1	21.20	–	8.46	4.97	1.518
1	2	1	Model 2	21.20	− 33.32	14.16	–	2.483
1	2	1	Model 3	21.20	–	9.70	8.30	**0.043**
1	2	1	Model 4	21.20	− 5.45	5.39	–	2.342
1	2	1	Model 5	21.20	–	3.76	7.77	3.939
1	2	1	Model 6	21.20	N/A	N/A	N/A	N/A
1	2	1	Model 7	21.20	–	5.94	2.30	0.665
1	2	5	Model 1	21.20	–	6.18	3.81	2.102
1	2	5	Model 2	21.20	− 22.03	10.67	–	3.781
1	2	5	Model 3	21.20	–	10.03	8.91	**0.100**
1	2	5	Model 4	21.20	− 5.45	5.39	–	8.567
1	2	5	Model 5	21.20	–	4.17	6.31	10.821
1	2	5	Model 6	21.20	N/A	N/A	N/A	N/A
1	2	5	Model 7	21.20	–	4.47	2.39	1.330
1	2	10	Model 1	21.20	–	5.36	5.02	1.944
1	2	10	Model 2	21.20	− 13.83	10.78	–	4.359
1	2	10	Model 3	21.20	–	10.56	9.43	**0.152**
1	2	10	Model 4	21.20	− 3.59	4.42	–	20.468
1	2	10	Model 5	21.20	–	4.17	6.31	25.650
1	2	10	Model 6	21.20	N/A	N/A	N/A	N/A
1	2	10	Model 7	21.20	–	3.47	3.44	0.990
1	3	1	Model 1	20.4	–	7.26	2.33	0.760
1	3	1	Model 2	20.4	− 45.00	8.74	–	1.341
1	3	1	Model 3	20.4	–	8.82	10.41	**0.073**
1	3	1	Model 4	20.4	− 4.07	5.29	–	0.896
1	3	1	Model 5	20.4	–	5.54	1.57	0.674
1	3	1	Model 6	20.4	N/A	N/A	N/A	N/A
1	3	1	Model 7	20.4	–	6.72	1.36	0.497
1	3	5	Model 1	20.4	–	7.20	2.77	1.365
1	3	5	Model 2	20.4	− 25.63	9.22	–	1.443
1	3	5	Model 3	20.4	–	9.05	11.73	**0.103**
1	3	5	Model 4	20.4	− 4.07	5.29	–	3.365
1	3	5	Model 5	20.4	–	5.54	1.57	1.782
1	3	5	Model 6	20.4	N/A	N/A	N/A	N/A
1	3	5	Model 7	20.4	–	6.75	1.28	1.375
1	3	10	Model 1	20.4	–	6.80	4.10	1.358
1	3	10	Model 2	20.4	− 16.55	10.40	–	1.045
1	3	10	Model 3	20.4	–	9.55	12.84	**0.105**
1	3	10	Model 4	20.4	− 4.07	5.29	–	4.235
1	3	10	Model 5	20.4	–	5.54	1.57	4.975
1	3	10	Model 6	20.4	N/A	N/A	N/A	N/A
1	3	10	Model 7	20.4	–	5.95	2.21	1.230

*T = *E*/*R* is an embedment ratio, *D* = *H*/*R* is a layer depth ratio, *b* is a mass ratio. N/A indicates no real number solution is found.

Significant values are in bold.

Furthermore, Model 3 shows relatively broader and more accurate adaptability than other model candidates in simulating the target soil-foundation system. Therefore, Model 3 is selected as a generic model in this study to simulate the dynamic interactions between the cylindrical foundation and the soil medium. All the following results are analyzed using Model 3.

## Model validations on frequency–response curves

**Figure 3:**
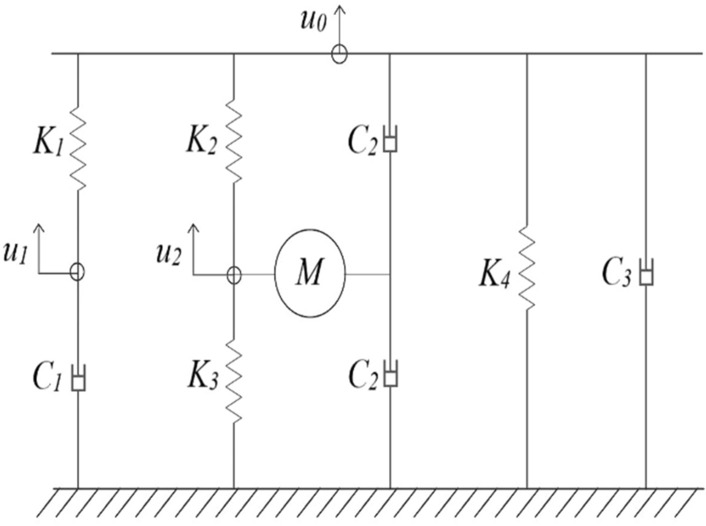
The lumped-parameter model by Wolf and Paronesso^25^ (1992).

This section aims to verify the frequency–response curves calculated by the generic model and introduce related research considering cylindrical foundations overlying uniform soil on a rigid base. Figure 3 shows that the Wolf and Paronesso model^25^ uses 3 degrees of freedom and eight parameters to simulate the SSI system (as shown in Fig. 1). The frequency–response curves and the peak response of the foundation by the generic model are compared and validated to those by the existing model. Wolf and Paronesso model^25^ was applicable as the dimensionless frequency *a*_0_ ≦ 4.1 for surface foundations and as *a*_0_ ≦ 2.5 for embedded foundations. By adopting the theoretical impedance and least-error analysis, this research breaks through the frequency limitation of the Wolf and Paronesso model^25^. This work effectively extends the dimensionless frequency (*a*_0_) from 4.1 to 6.2 for surface foundations and 2.5 to 3 for embedded foundations, as shown in Table. 5.

**Table 5:** Comparisons of simulation parameters between the existing and proposed models.

Model name	Embedded depth ratio	Layer depth ratio	Maximum dimensionless frequency
Wolf and Paronesso model^25^	0	1–4	4.1
	1	2–3	2.5
Proposed model	0	0.25–10	6.2
	0.25–2	0.5–10	3

### Surface cylindrical foundations

The generic model uses the impedance function from Tassoulas and Kausel^31^ (1983) to generate lump-parameter models for simulating the target stratum. Based on the calculation of the optimal equivalent model, numerous simplified models are generated using equivalent principles, and then the optimal equivalent model is obtained.

**Figure 4:**
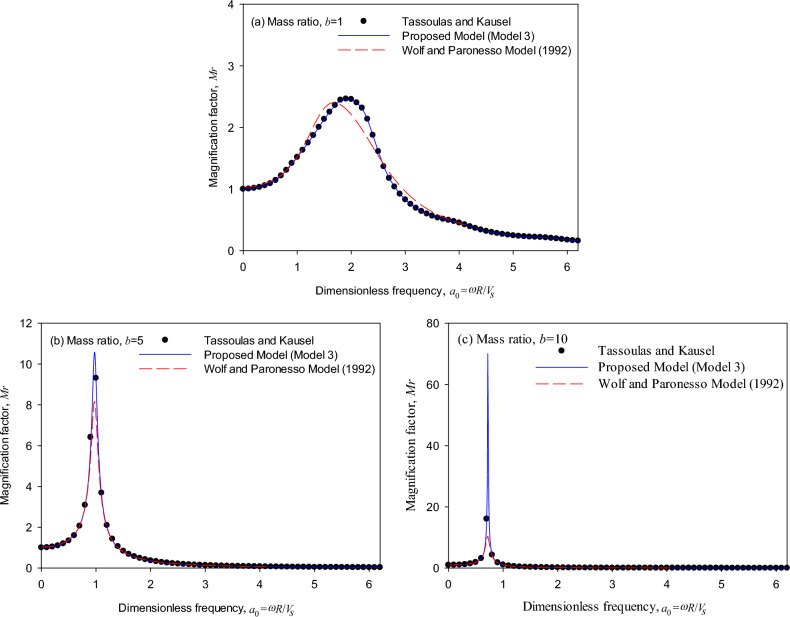
Dynamic response of surface foundations (*T* = 0, *D* = 2): (**a**) mass ratio *b* = 1, (**b**) mass ratio *b* = 5, (**c**) mass ratio *b* = 10.

Figure 4 shows the relationship of frequency versus response for a cylindrical surface foundation (*T* = 0, *D* = 2). The generic model generates results consistent with the dynamic magnification factors obtained by the theoretical solutions in the cases where the mass ratio increases from 1, 5 to 10, which validates the accuracy of the generic model. It is particularly noted in Fig. 4a that as the mass ratio *b* is small (i.e., *b* = 1), the generic model simulates the dynamic magnification factor at 1 < *a*_0_ < 4 more precisely than Wolf and Paronesso model^23^. Furthermore, Fig. 4 demonstrates that the generic model simulates more precisely than the existing model in the peak responses as the mass ratio varies from 1, 5 to 10. The reason for frequency–response differences might be that the curve-fitting method by Wolf and Paronesso model^25^ used a different error weighting function to approach the theoretical impedance. In contrast, the generic model considers the effect of mass ratios to approach the frequency–response curve.

### Embedded cylindrical foundations

The generic model uses the impedance function by Tassoulas^32^ (1981) to construct simplified models for simulating target stratum. There are 30 given frequency points of *a*_0_. A series of simplified models are generated using equivalent principles; then, the optimal equivalent model is obtained by Eq. (11).

**Figure 5:**
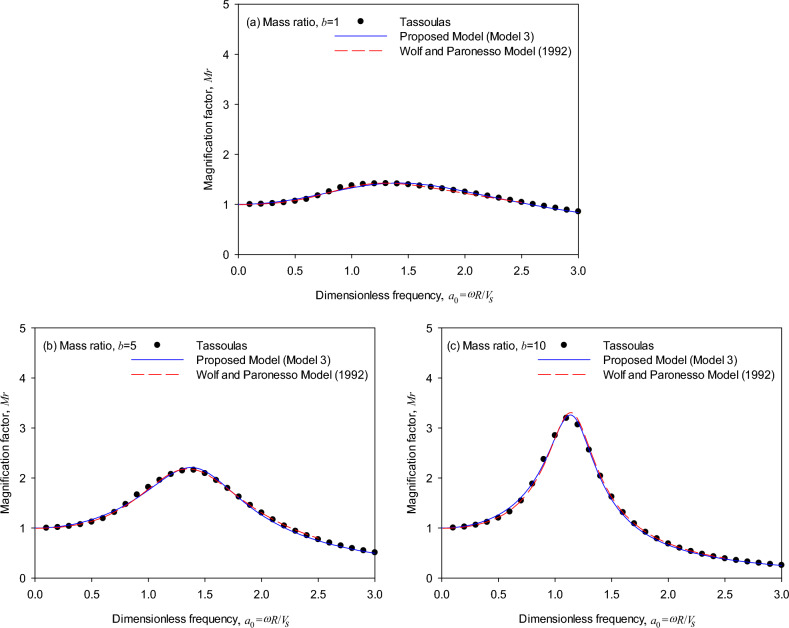
Dynamic response of embedded foundations (*T* = 1, *D* = 2): (**a**) mass ratio *b* = 1, (**b**) mass ratio *b* = 5, (**c**) mass ratio *b* = 10.

Figure 5 shows the trend of the frequency-response relationship for the cylindrical foundation (*T* = 1, *D* = 2). The generic model and the Wolf and Paronesso model^25^ produce results consistent with the magnification factors calculated by the theoretical impedances. It is worth mentioning that the generic model adopting only three parameters performs more effectively than the existing model.

**Figure 6:**
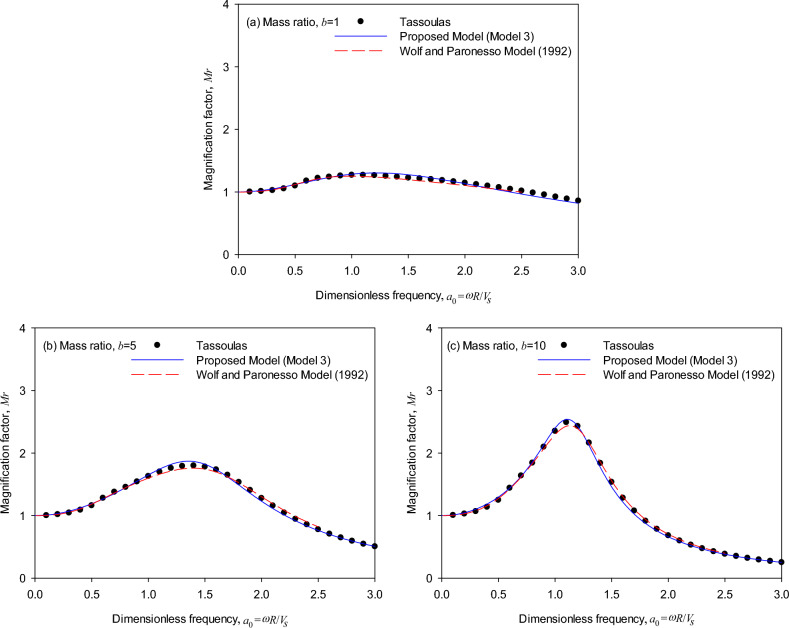
Dynamic response of embedded foundations (*T* = 1, *D* = 3): (**a**) mass ratio *b* = 1, (**b**) mass ratio *b* = 5, (**c**) mass ratio *b* = 10.

Figure 6 shows the trend of the frequency-response relationship for the cylindrical foundation (*T* = 1, *D* = 3). It is observed by compared with Fig. 5 as the layer depth ratio (*D*) increases, the generic model also produces results consistent with the magnification factors calculated by the theoretical impedances as the mass ratio varies from 1, 5 to 10, which validates the accuracy of the generic model. On the other hand, it is observed in Fig. 6 that the Wolf and Paronesso model^25^ seems to underestimate the peak responses in all mass ratios slightly.

## Model validations on resonant responses

This section focuses on validating the resonant responses obtained by the generic model (i.e., Model 3), which is significant for engineering applications to avoid the coincidence of the forcing frequency with the natural frequency of the soil-foundation system. The resonant response of the foundation consists of the maximum magnification factor and the corresponding frequency. The discontinuity of the adopted impedance function shows discrete characteristics^18^; therefore, catching the peak response of the foundation between discrete frequency points appears necessary and significant. The five-point interpolation technique^30^ is adopted to catch the peak responses of the theoretical solution by Tassoulas and Kausel^31^ (1983) and Tassoulas^32^ (1981). In addition, as Wolf and Paronesso model^25^ (1992) calculates the dynamic response by evaluating the torsional impedance functions based on its model parameters, the five-point interpolation approach^30^ is also applied to Wolf and Paronesso model^25^ to calculate the peak displacements. However, the peak displacements using the generic model do not perform the five-point interpolation approach^30^ because the three model parameters vary with the mass ratio. To catch the approximate peak response for the foundation, this study adopts the impedance function with expanded frequencies (i.e., the interval of *a*_0_ is 0.1) by a standard third-order interpolation function. Thus, the analyzed peak reactions of the foundation can be directly selected among the calculated discrete responses.

### Surface cylindrical foundations

**Figure 7:**
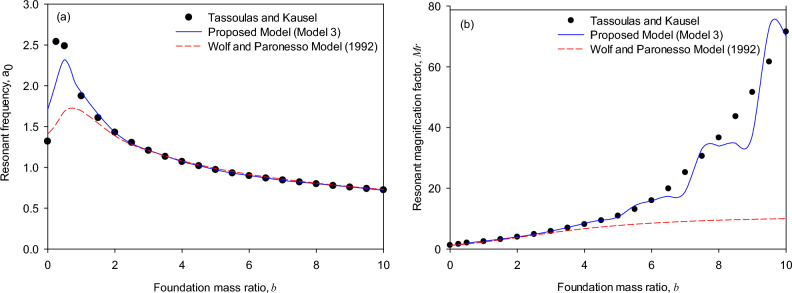
Resonant response of surface foundations (*T* = 0, *D* = 2) overlaying on a soil stratum on rigid base: (**a**) resonant frequency, (**b**) resonant magnification factor.

Figure 7a (*T* = 0, *D* = 2) shows that the resonant frequencies of the torsional foundation response calculated by both the generic model and the Wolf and Paronesso model^25^ are consistent well with the theoretical solutions as the torsional mass ratios of the foundation are in the range (*b* = 2 to 10). On the other hand, it is comparatively observed that the generic model and the Wolf and Paronesso model^25^ could underestimate the resonant frequencies as the torsional mass ratios of the foundation are small (*b* = 0 to 2). In contrast, the generic model evaluates the resonant frequencies more accurately than the existing model.

Figure 7b (*T* = 0, *D* = 2) shows that resonant magnification factors of the torsional foundation response calculated by the generic model are consistent well with the theoretical solutions as the torsional mass ratio of the foundation varies in the range from 0 to 10. In addition, it is comparatively observed that the generic model simulates more precisely. At the same time, the Wolf and Paronesso model^25^ significantly underestimates the resonant magnification factors of the torsional foundation responses as the torsional mass ratios of the foundation are larger (*b* = 3 to 10). The reasons that caused these differences in the resonant magnification factor might be that the peak magnification factor is fairly sensitive to large mass ratios, especially as the layer depth ratio of the soil stratum is small, and that the generic model adopts the optimal equivalent model which carefully considers the effect of each single mass ratio on the peak response.

Note that Fig. 7(b) (*T* = 0, *D* = 2) shows the results for a relatively small layer depth ratio of the soil stratum. A remark is that the analyzed results of the resonant magnification factors simulated by the generic model show agreement with the theoretical solutions and are consistent with the whipping effect of the soil-structure interaction^33^.

### Embedded cylindrical foundation

**Figure 8:**
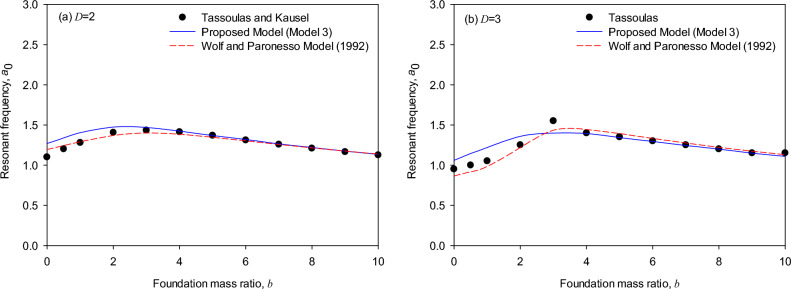
Resonant frequency of embedded foundations (*T* = 1): (**a**) layer depth ratio *D* = 2, (**b**) layer depth ratio *D* = 3.

Figure 8a (*T* = 1, *D* = 2) shows that resonant frequencies of the torsional foundation response calculated by both the generic model and the Wolf and Paronesso model^25^ are consistent well with the theoretical solutions as the torsional mass ratios of the foundation are in the range (*b* = 4 to 10). On the other hand, as the torsional mass ratios of the foundation are in the range (*b* = 0 to 4), it is comparatively observed that the generic model and the Wolf and Paronesso model^25^ could respectively overestimate and underestimate the resonant frequencies. Whereas the Wolf and Paronesso model^23^ evaluates the resonant frequencies slightly more accurately than the generic model as mass ratios are small (*b* = 0 to 4). Figure 8b (*T* = 1, *D* = 3) shows that as the torsional mass ratios of the foundation are in the larger range (*b* = 4 to 10), resonant frequencies of the torsional foundation response calculated by the generic model are consistent well with the theoretical solutions while the Wolf and Paronesso model^25^ slightly overestimates. However, as the torsional mass ratios of the foundation are in the smaller range (*b* = 0 to 4), the generic model slightly overestimates the resonant frequencies while the Wolf and Paronesso model^25^ slightly underestimates resonant frequencies. Generally, the generic model and the Wolf and Paronesso model^25^ simulate the resonant frequencies in a way that approaches theoretical solutions as the mass ratio increases.

**Figure 9:**
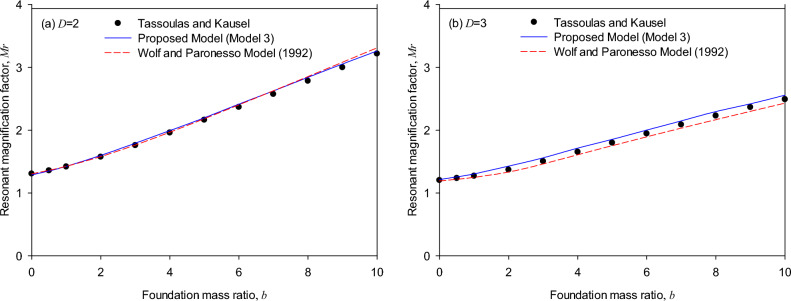
Resonant magnification factor of embedded foundations (*T* = 1): (**a**) layer depth ratio *D* = 2, (**b**) layer depth ratio *D* = 3.

Figure 9 (*T* = 1, *D* = 2 and 3) shows that resonant magnification factors of the torsional foundation response simulated by the generic model and the Wolf and Paronesso model are both consistent well with the theoretical solutions as the torsional mass ratio of the foundation varies in the range from 0 to 10. In addition, it is comparatively observed in Fig. 9b that the generic model outperforms the existing model in terms of the Wolf and Paronesso model^25^ slightly underestimating the resonant magnification factors of the torsional foundation at all mass ratios considered (*b* = 0 to 10). The generic model performs better in the resonant magnification factor because the resonant magnification factor of the theoretical solution is precisely detected by the five-point interpolation method. Moreover, the generic model considers the effect of mass ratios on the dynamic magnification factor. The generic model seems to estimate better the resonant magnification factor for surface and embedded foundations.

## Multiple target approach (MTA) for practical applications

The STA illustrated in the previous section is limited to the application for a single mass ratio. In light of STA’s limitations, a multiple target approach (MTA) is developed, as listed in Supplementary Information-SCP, to enhance the engineering application of the proposed model. This section aims to provide reference charts for engineering use to build the generic model quickly. The Wolf and Paronesso model^25^ was developed in the 1990s. It was a pioneering study at that time, but the model element was complex, and there was no relevant parametric chart for engineering applications. Therefore, this study further proposes the MTA to find a generic model and consider a broader range of soil-foundation parameters to build related parametric charts, making the simplified model easier to apply in engineering.

### Dimensionless parametric charts

In this subsection, parametric charts are established based on the procedure of MTA listed in Supplementary Information-SCP, considering multiple mass ratios. Consider that the maximum dimensionless frequency is 3 with a discrete interval of 0.1, then the number of dimensionless frequency (*NF*) is 30. Assume that a group of mass ratios (*b* = 0.5,1,2,3,4,5) is assigned, then the number of multiple mass ratios (*NB*) is 6. Applying Supplementary Information-SCP, 7 × *NB* × *NF* (i.e., 7 × 6 × 30) sets of simplified models are generated, and the model parameters of the optimal equivalent model are obtained at each combination of the embedment depth ratio (*T*), and the layer depth ratio (*D*). The embedment depth ratios are *T* = 0, 0.25, 0.5, 1, 1.5, and 2. The minimum layer depth ratio is defined as *D*_*min*_ = *T* + 0.25, and the maximum layer depth ratio is defined as *D*_*max*_ = 10 with an integral interval of 1 from the minimum. After analyzing the optimal equivalent model for multiple mass ratios considering various soil and foundation parameters, the analyzed results determine that the optimal equivalent model is solely the model candidate (Model 3). The result that Model 3 is the optimal simplified model among the 7 model candidates is consistent with that in Tables 3 and 4. Recall that Model 3 is selected as the generic model for generating corresponding dimensionless charts. In addition, Table 5 shows that the proposed model in this research considers a broader range of main soil-foundation parameters than the Wolf and Paronesso model^25^ (1992).

**Figure 10:**
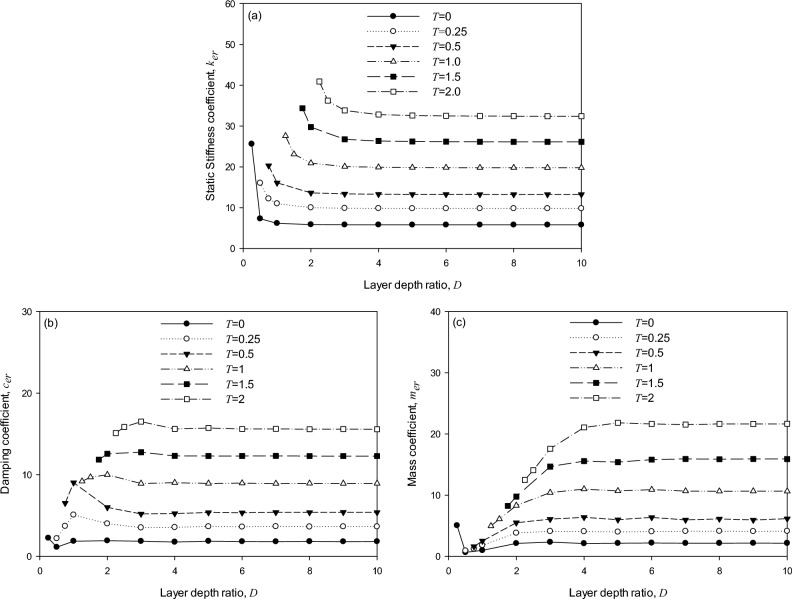
Dimensionless parametric charts for creating the generic model: (**a**) stiffness coefficient, (**b**)damping coefficient, and (**c**)mass coefficient.

Furthermore, Fig. 10a–c are generated as dimensionless charts of the static stiffness coefficient ($k_{er}$), damping coefficient ($c_{er}$), and mass coefficient ($m_{er}$), respectively, for the genetic model. Those referenced values in the chars correspond to the referenced scenarios to compute the dynamic responses of foundations. Error analysis of applying the charts will be substantially introduced in the next subsection, followed by an illustrative example for practical engineering application.

### Error analysis and applicable scope

This subsection compares the errors by different approaches and gives suggestions for application. The STA follows the process described in the previous section to build the optimal model for a single mass ratio. The MTA applies the dimensionless parametric charts given in Fig. 10 to create the optimal model quickly. The optimal models generated by STA and MTA may have different performances in simulating the target system. Thus, it is essential to investigate the difference between the two approaches. Eqs. (12) and (13) define a relative error index for the STA, $\varepsilon_{STA}$ and for the MTA, $\varepsilon_{MTA}$, respectively.

12 $$\varepsilon_{STA} = \sqrt {\frac{{\mathop \sum \nolimits_{i = 1}^{NB} \mathop \sum \nolimits_{j = 1}^{NF} \left( {M_{system\,i,j} - M_{STA\,i,j} } \right)^{2} }}{{\mathop \sum \nolimits_{i = 1}^{NB} \mathop \sum \nolimits_{j = 1}^{NF} \left( {M_{system\,i,j} } \right)^{2} }}}$$

13 $$\varepsilon_{MTA} = \sqrt {\frac{{\mathop \sum \nolimits_{i = 1}^{NB} \mathop \sum \nolimits_{j = 1}^{NF} \left( {M_{system\,i,j} - M_{MTA\,i,j} } \right)^{2} }}{{\mathop \sum \nolimits_{i = 1}^{NB} \mathop \sum \nolimits_{j = 1}^{NF} \left( {M_{system\,i,j} } \right)^{2} }}}$$

where $M_{system\,i,j}$, $M_{STA\,i,j}$, and $M_{MTA\,i,j}$ indicate the magnification factors calculated respectively using SASSI^30^ program, the STA, and the MTA for the *ith* mass ratio and *jth* dimensionless frequency ratio.

The assumption of multiple mass ratio combination is* b* = 0.5, 1, 2, 3, 4, and 5. Thus, based on the maximum and minimum of multiple mass ratios, the comparison of error index that corresponds to mass ratio *b* = 0.5, 1, 2.5, and 5 are selected. It is noted that each relative error index corresponds to each combination of the embedment depth ratio (*T*), the layer depth ratio (*D*), and the mass ratio (*b*).

**Figure 11:**
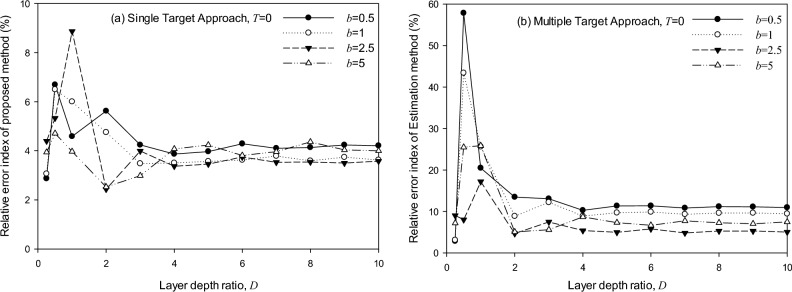
Error index analysis for surface cylindrical foundation: (**a**) STA, (**b**) MTA.

Figure 11 shows the error index, by Eqs. (12) and (13), of surface foundation considering various soil-foundation conditions (*T* = 0, *D* = 0.5–10, *b* = 0.5, 1, 2.5, and 5). Remarks are made as follows:

- The STA shows a good agreement with the SASSI^30^ solution and a precision higher than 91.1%, as the maximum error occurs at *T* = 0, *D* = 1, and *b* = 2.5.
- The MTA shows a precision of 42.1% ~ 97.1% with the numerical solutions, as the maximum error occurs at *T* = 0, *D* = 0.5, and *b* = 0.5. However, adjusting the application range to 4≦*D*≦10 and 1≦*b*≦5 is suggested to demonstrate a precision higher than 90%.

**Figure 12:**
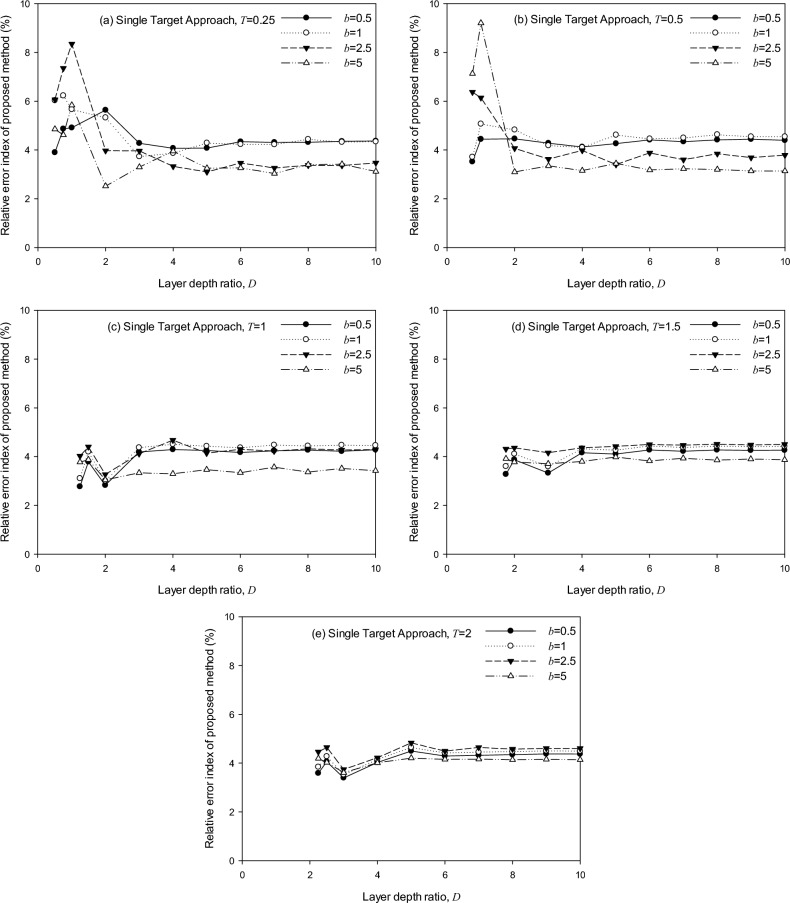
Error index by the generic model from STA for embedded cylindrical foundation regarding various embedment ratios: (**a**) *T* = 0.25, (**b**) *T* = 0.5, (**c**) *T* = 1, (**d**) *T* = 1.5, (**e**) *T* = 2.

**Figure 13:**
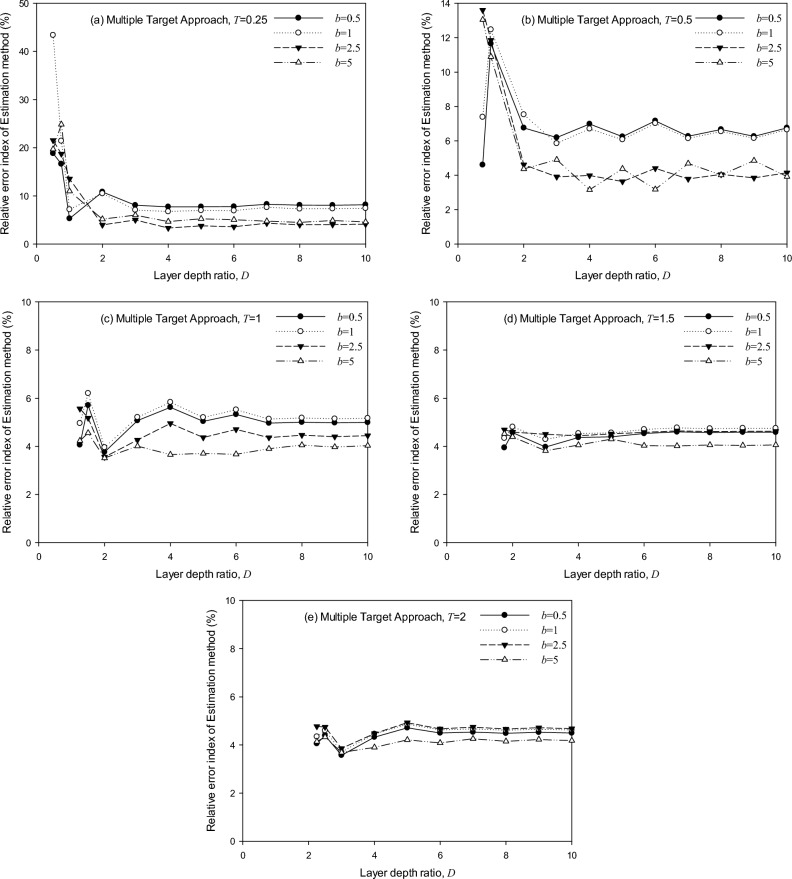
Error index by the generic model from MTA for embedded cylindrical foundation regarding various embedment ratios: (**a**) *T* = 0.25, (**b**) *T* = 0.5, (**c**) *T* = 1, (**d**) *T* = 1.5, (**e**) *T* = 2.

Figures 12 and 13 show the error index by Eqs. (12) and (13), of embedded foundations, considering various soil-foundation conditions. (*T* = 0.25, 0.5, 1, 1.5, and 2, *D* = 0.75 ~ 10, *b* = 0.5, 1, 2.5, and 5). Remarks are made as follows:

- The STA shows a good agreement with the SASSI^30^ solution and a precision higher than 90.8%, as the maximum error occurs at *T* = 0.5, *D* = 1, *b* = 5.
- The MTA shows a precision of 56.6–96.8% with the SASSI^30^ solutions, as the maximum error occurs at *T* = 0.25, *D* = 0.5, and *b* = 1. Adjusting the application range to 2 < *D*≦10 and 0.5≦*b*≦5 is suggested to reach a precision higher than 90%.

### Illustrative example

Consider a rigid cylindrical foundation embedded in uniform soil on a rigid base, as shown in Fig. 1. The generic model (i.e., Model 3 shown in Fig. 2) is developed herein to simulate the interactions between the foundation and the soil medium. The properties of the target system are described as follows:

- Foundation geometry: a radius of the foundation, *R* = 2.5 m, and an embedment depth, *E* = 2.5 m.
- Soil properties: Poisson ratio, *ν* = 0.33; damping ratio, *ζ* = 0.05; mass density, *ρ* = 2000Mg/m^3^; shear-wave velocity, *V*_*s*_ = 400 m/s; a thickness of the soil stratum, *H* = 12.5 m.

For the target system considered, the embedment ratio *T* = 2.5/2.5 = 1 and the layer depth ratio *D* = *E*/*R* = 12.5/2.5 = 5. Subsequently, the dimensionless coefficients $k_{er}$, $c_{er}$, and $m_{er}$ are directly obtained by querying Fig. 10, i.e. $k_{er}$ = 19.83, $c_{er}$ = 10.68, and $m_{er}$ = 8.94. Hence, the model parameters for the soil-foundation system can be evaluated through Eq. (1), as shown below.

14 $$K_{er} = k_{er} GR^{3} = {99},{15}0 \times 10^{6} \,{\text{kN}}\,{\text{m}}$$

15 $$C_{er} = c_{er} \;\rho V_{s} R^{4} = {333},{75}0\,{\text{kN}}\,{\text{s/m}}$$

16 $$M_{er} = m_{er} \,\rho R^{5} = {1},{746},0{94}\,{\text{Mg}}\,{\text{m}}^{{2}}$$

The process above may show the effectiveness of the parametric charts developed for engineering applications.

## Conclusion

A systematic method is developed to investigate lump-parameter models for various homogeneous soil strata on rigid base, considering the dynamic interaction behavior of soil-foundation systems. The systematic method proposes seven simplified models comprising springs, dampers, and lumped mass. This research utilizes the proposed method to adaptively identify a generic model to investigate the dynamic response of a cylindrical foundation embedded in uniform soil on rigid base under torsional vibrations. The generic model simulates the foundation response considering embedment depth ratios, layer depth ratios, and mass ratios. The validation of the generic model to simulate the dynamic frequency-magnification curve and resonant behavior has been confirmed. The generic model effectively overcomes the limitation of the Wolf and Paronesso model about the upper-frequency limit in frequency–response curves. In addition, the existing model underestimates the resonant magnification factors when the foundation mass ratio increases.

Moreover, the proposed model has broader applicability than the Wolf and Paronesso model and has also achieved a precision higher than 90% within the suggested application range. Dimensionless parametric charts are proposed to estimate the model parameters readily and to efficiently build the generic model in the time domain for the dynamic analysis of cylindrical foundations under torsional load. The research results showed the limitation of the existing model may be improved, as follows: the foundation embedment deeper than the foundation radius (*R*), the depth of the soil layer greater than 3*R*, and the dimensionless frequency ratio larger than 2.5.

This research developed a generic simplified model with better practical applications than the Wolf and Paronesso model. Additionally, this study provides the single target approach (STA) and the multiple target approach (MTA) to find the optimal equivalent models for simulating target soil-foundation systems. The STA establishes an optimal model for a target system vibrated with a fixed mass. The MTA shows excellent potential in engineering applications regarding various soil-foundation conditions. The generic model may be applied efficiently and accurately to simulate the dynamic torsional responses for the cylindrical foundation embedded in uniform soils on rigid base. For more complicated layers of soils, additional discrete-element modelings may be further extended to enhance the capabilities of the generative models.

### Supplementary Information

Supplementary Information.

## Data Availability

All data generated or analyzed during this study are included in this published article.
