# Supplementary material for: Dynamic analysis of cylindrical foundations under torsional loading via generic discrete-element models simulating soil stratum
Source: Sci Rep. 2023 Nov 6;13:19163. doi: 10.1038/s41598-023-46046-7 (PMC10628243; doi:10.1038/s41598-023-46046-7)
Supplement: Supplementary file 1 — Supplementary Information. [file 41598_2023_46046_MOESM1_ESM.pdf]

Dynamic Analysis of Cylindrical Foundations under Torsional Loading via Generic Discrete-Element Models Simulating Soil Stratum

Shi-Shuenn Chen[1,*,+], **Chi-Jou Kao**[1,+], **Jun-Yang Shi** [2,+]

[1]Department of Civil and Construction Engineering, National Taiwan University of Science and Technology, Taipei, 106, Taiwan
[2]Department of Civil and Environmental Engineering, National University of Kaohsiung, Kaohsiung, 811, Taiwan
[*]sschen@mail.ntust.edu.tw
[+]these authors contributed equally to this work

Supplementary Information-SCP

Overall Introduction

Supplementary Information-SCP describes the calculation procedure of the systematic approach considering multiple mass ratios. As the torsional impedance function for a target soil-foundation system is given, the model parameters of all seven simplified models can be obtained. Taking Model 3 as an example among seven model candidates, the model parameters of model 3 are derived by applying Manuscript Eqs. (4) to (6) as follows:

$$k_{er}=\frac{K_{er}}{GR^3},\ m_{er}=\frac{k_{er}(1+a_0^2c_r^2\text{-}2k_r+k_r^2)}{a_0^2(1\text{-}k_r)},\ c_{er}=\frac{k_{er}(1+a_0^2c_r^2\text{-}2k_r+k_r^2)}{a_0^2c_r} \tag{E1}$$

Assuming that the impedance function of the foundation is considered to be NF frequencies, then the systematic approach establishes 7× NF simplified models when considering single mass ratio. Analogously, the systematic approach establishes 7 × NB × NF simplified models while considering NB multiple mass ratios. In terms of generalizing the effects of mass ratios, Supplementary Information-SCP introduces the optimal equivalent model considering multiple mass ratios, which is beneficial to generate parametric charts for potential engineering application.

Optimal equivalent model for multiple mass ratio

The steps of using the systematic approach to find the optimal equivalent model for multiple mass ratios are shown in the following:

1. The referenced impedance function and corresponding magnification factors of the foundation in a soil-foundation system considering various embedment depth ratio (T), the layer depth ratio (D), and mass ratios (b) is obtained by SASSI, a computer program developed by Lysmer et al. Assume NB amounts of multiple mass ratios, and NF frequencies are considered, then NB x NF sets of magnification factors will be established as the matrix of system magnification factor, shown as $M_{System}=\begin{bmatrix} M_{1,1} & \cdots & M_{1,NF} \\ \vdots & \ddots & \vdots \\ M_{NB,1} & \cdots & M_{NB,NF} \end{bmatrix}$.
2. Based on the procedure as illustrated in Manuscript Eqs. (4) to (6), and using the referenced impedance functions analyzed by SASSI, three model parameters of the simplified model are generated using equivalent principles in every combination of 7 model candidates, NB mass ratios, and NF frequency points. Therefore, 7 x NB x NF sets of coefficients of the simplified models are calculated sequentially and expressed in terms of matrix form as $C_{model\_k}=\begin{bmatrix} C_{1,1} & \cdots & C_{1,NF} \\ \vdots & \ddots & \vdots \\ C_{NB,1} & \cdots & C_{NB,NF} \end{bmatrix}$ for k=1,2,3,4,5,6,7; k indicates the No. of model candidates (as shown in Manuscript

Fig. 2 and Manuscript Table 2); k is identically defined if it is mentioned again in the rest of the following. It is noted that the C_{model_k} matrix corresponds to 7 model candidates. Each $C_{i,j}$ in the C_{model_k} matrix indicates a vector, either $[k_{er_{i,j}} \quad c_{er_{i,j}} \quad m_{er_{i,j}}]$ or $[k_{er_{i,j}} \quad c_{er_{i,j}} \quad k_{d_{i,j}}]$, depending on model parameters of the specific model candidate, shown as Manuscript Table 2. $k_{er_{i,j}}$, $k_{d_{i,j}}$, $c_{er_{i,j}}$,and $m_{er_{i,j}}$ respectively represents the static stiffness, dynamic stiffness, damping, and mass coefficient of the simplified model at mass ratio $b=i$ and dimensionless frequency $a_0=j$.

3. Use the three model parameters of every element $C_{i,j}$ in each C_{model_k} matrix to sequentially create the corresponding matrix of model magnification factor, shown as $M_{model\_k}=\begin{bmatrix} M_{1,1} & \cdots & M_{1,NF} \\ \vdots & \ddots & \vdots \\ M_{NB,1} & \cdots & M_{NB,NF} \end{bmatrix}$ for k=1,2,3,4,5,6,7. It is noted that the M_{model_k} matrix corresponds to 7 model candidates. Calculate the matrix of weighted relative error ε_{model_k} sequentially, i.e.,

$$\varepsilon_{model\_k}=\left\{(M_{system_{i,j}}\text{-}M_{model_{i,j}})^2\,(P_{i,j})\right\} \text{ for k=1,2,3,4,5,6,7} \qquad \text{(E2)}$$

Where $P_{i,j} = M_{system_{i,j}}$ and it is noted that the ε_k matrix corresponds to 7 model candidates and can be expressed as

$$\varepsilon_{model\_k}=\begin{bmatrix} \varepsilon_{1,1} & \cdots & \varepsilon_{1,NF} \\ \vdots & \ddots & \vdots \\ \varepsilon_{NB,1} & \cdots & \varepsilon_{NB,NF} \end{bmatrix}.$$

4. Apply equation (E2) to calculate sequentially the sum of weighted relative error for seven model candidates $\varepsilon_{sum\_model\_k}$, and then the model candidate corresponding to the minimum of $\varepsilon_{sum\_model\_k}$ is determined as the optimal equivalent model considering multiple mass ratios.

$$\varepsilon_{sum\_model\_k}=\sum_{i=1}^{NB}\sum_{J=1}^{NF}\varepsilon_{i,j} \text{ for } k\text{=1,2,3,4,5,6,7} \qquad \text{(E3)}$$
